# Supplementary material for: Attitudes towards Monkeypox vaccination and predictors of vaccination intentions among the US general public
Source: PLoS One. 2022 Dec 1;17(12):e0278622. doi: 10.1371/journal.pone.0278622 (PMC9714903; doi:10.1371/journal.pone.0278622)
Supplement: Attachment — Submitted filename: Rebuttal_Monkeypox_PONE.docx [file pone.0278622.s001.docx]

# Rebuttal Letter

We would like to thank the reviewers for their comments and suggestions on our manuscript. We have addressed each comment in the letter below, our answers are marked in blue. We have also indicated where changes in the manuscript were made.

On behalf of the authors,

Dr. Maike Winters

## Reviewer 1

1. Authors consider to assess previous “COVID-19 vaccination status” as the predictor (only those > 45 y.o. asked for previous smallpox vaccination). In the case of COVID-19, previous influenza vaccination was studied as the predictor due to the similar characteristics of these disease. Why are the reasons behind this? What about the COVID-19 vaccination coverage in the US?

*Thank you for these questions. Given how polarizing the COVID-19 pandemic and COVID-19 vaccination has been, especially in the US where our study takes place, we hypothesized that COVID-19 vaccination status could be a predictor of vaccination attitudes and intentions of other vaccines. The recently reported substantial decrease in routine immunization coverage around the world is an example of this: even when vaccination services resumed around the world in 2021, the decrease in coverage continued (See UNICEF data:* [*https://data.unicef.org/topic/child-health/immunization/*](https://data.unicef.org/topic/child-health/immunization/) *). More negative attitudes towards the COVID-19 vaccines (and thus, likely not being vaccinated against COVID-19) could therefore also influence Monkeypox vaccination intentions.*

2. Results from questionnaire validation should be presented per-item.

*Thank you for this comment and apologies if there is some confusion around the Vaccine Trust Indicator. This scale has been validated in previous surveys, the manuscript of which is currently under submission at a peer-reviewed journal. The aim of our study was therefore not to validate it, but merely to use it as a consolidated predictor of vaccine acceptance.*

3. Why occupation is not included in the patients characteristics?

*We have not included occupation in the characteristics description, because we have not asked about this in the survey. Our socio-demographic characteristics include age, gender, education, race and ethnicity. Especially education correlates strongly with occupation - to keep the survey length manageable we opted to only ask about education.*

4. In Education variables; what are the differences between college and some college. And why education was not assessed as the predictor?

*Thank you for this question. The ‘Some college’ category means that people enrolled in college, but did not graduate. The ‘College’ category means that people have graduated from college. We did not find a statistically significant association between education and Monkeypox vaccination intention, which is why we have not separately reported it in table 4. However, the adjusted associations shown in table 4 are adjusted for education, as reported in the table’s footnotes.*

5. Line 252-257. Regarding the vaccination safety. This may be of interest to authors to compare the results obtained from countries in Asia, Africa, and South America. Rosiello et al 2021 Narra J 1(3):e55 doi: 10.52225/narra.v1i3.55

*Thank you for sharing this interesting paper. We have added a sentence in the Discussion:*

*‘While our study looked at risk perception of the disease, the perceived risk of side effects and perceptions of vaccine efficacy can influence vaccination intentions as well (Rosiello 2021).’ (Lines 288-290)*

6. “healthy lifestyle” what is the concept that vaccination could be a part of healthy lifestyle. Kindly explain.

*This is part of the Vaccine Trust Indicator, a 6-item scale that has recently been validated (see answer to question 2). The idea of this statement around ‘healthy lifestyle’ is the notion that people have more positive attitudes towards vaccination (and are more likely to be vaccinated) when they perceive vaccination to be part of their attempts to live a healthy lifestyle.*

## Reviewer 2

Thanks for the invitation to review this interesting and timely manuscript.

In the current study, Maike Winters et al. conducted a KAP study on the monkeypox vaccination using a sample of adults in the US.

The major results pointed to relatively high prevalence of vaccine hesitancy or rejection besides the poor knowledge about the emerging disease in about half of the study sample.

Overall, the study is well-written with a clear description of the methodology, and clear presentation of the results. However, the Introduction and Discussion can benefit from a thorough literature review besides the need to mention the potential study limitations.

*Thank you for these nice words. We have answered your questions and comments below.*

Minor points:

1. In line 35: it is recommended not to start a sentence with a number “46% of respondents…”

*Thank you for noticing this, we have changed the order of the sentence:*

*‘If recommended, 46% of respondents intended to get vaccinated against Monkeypox,...’ (lines 35,36)*

2. In the Introduction, the authors can benefit from the following recent references on the topic of monkeypox prevention through vaccination:

A. <https://narraj.org/main/article/view/90>

B. <https://www.mdpi.com/1999-4915/14/10/2155>

*These are great resources, thank you for sharing. As the introduction in our manuscript has gotten slightly outdated since we submitted it, we have now updated the section on the vaccines to reflect the latest evidence on real-world effectiveness and the vaccination strategy in the US. We have added the two papers in the references. Please see lines 66-86 in the Introduction.*

3. In the Introduction, the authors can benefit from the following recent reference on the role of MSM as a risk group and the importance of preventing stigma and discrimination towards this group:

A. <https://doi.org/10.1002/jmv.27913>

B. <https://doi.org/10.1002/jmv.27931>

*Thank you for these papers, we have added a sentence in the introduction on the importance of communication in preventing stigma and discrimination towards high risk groups, see lines 92-93:*

*‘In communication with the general public, special care should be taken to prevent stigmatization and discrimination towards high risk groups, such as MSM (Bragazzi 2022, Bragazzi 2022).’*

4. In the Methods, line 90: please provide more details regarding the online recruitment tool “CloudResearch”

*We have updated the Methods to include more information:*

*‘A representative sample of the U.S. adult population was recruited through market research company CloudResearch in early June 2022. Participants received a small monetary incentive after completing the survey.’ (lines 111-113)*

5. In the Methods, please provide more details regarding the approach used for sample size calculation.

*To calculate our sample size, we used a conservative number of estimated vaccination intentions among the US adult population of 50%, and a margin of error of 4%. This led to a sample size of 600, which we have increased to 800 to account for missing data. This has been updated in the manuscript, see lines 118-120.*

6. In the Discussion section, the authors can improve the discussion by checking the following recent references representing KAP studies addressing monkeypox:

A. <https://www.mdpi.com/2414-6366/7/7/135>

B. <https://www.mdpi.com/2227-9032/10/9/1722>

C. <https://www.mdpi.com/2076-0817/11/8/904>

*Thank you for sharing these papers. We have added references to the discussion. The Alsharani paper we cite in lines 248-250, as a comparison of general public’s knowledge outside of the US.*

*‘The low levels of self-assessed knowledge about Monkeypox are in line with general public’s Monkeypox knowledge elsewhere (Alsharani 2022) and indicate the need for clear communication about the outbreak.’*

*The papers by Sallam et al an Ricco et all are cited in the section where we discuss the importance of keeping health workers updated (lines 268-272):*

*‘To be able to communicate effectively about the outbreak and advise their patients, it would be important to target health workers specifically with ongoing updates around the outbreak and providing practical tools to communicate with their patients, as recent surveys (outside the US) have found that health worker knowledge about Monkeypox is suboptimal (Sallam 2022, Ricco 2022).’*

7. The authors should add a limitations section to address the potential caveats of the study. E.g. possible selection bias, measurement bias, lack of data on sexual orientation of the participants.

*Thank you for this comment. We have now added a section called ‘Strengths & Limitations’ to the Discussion (lines 305-316):*

***‘Strengths & Limitations***

*A major strength of our study was the ability to collect data from a representative sample of the US adult population, giving a rapid overview of knowledge gaps, vaccine attitudes and communication needs in a new outbreak. Limitations of the study include the fact that this is a cross-sectional survey – we cannot determine the direction of the associations we observed as reverse causality may have played a role. While we sampled using quotas to make the sample as representative as possible to the US adult population, it may be that certain groups were over- or under-sampled. We mitigated this by adding survey weights to the analysis. Given that the Monkeypox outbreak mainly affected MSM at the time of the survey, there may have been stigma and a selection bias in our study. This may have resulted in more negative attitudes towards vaccination. Lastly, we did not ask about the sexual orientation of the participants, which could have influenced vaccination intentions.’*

8. The supplementary table can be moved into the main text.

*We have moved the supplementary table to the main manuscript, where it is listed as Table 5.*

Thank you!
