# Supplementary material for: Attitudes towards Monkeypox vaccination and predictors of vaccination intentions among the US general public
Source: PLoS One. 2022 Dec 1;17(12):e0278622. doi: 10.1371/journal.pone.0278622 (PMC9714903; doi:10.1371/journal.pone.0278622)
Supplement: Attachment — Submitted filename: Rebuttal letter Revision 2-PLOSONE.docx [file pone.0278622.s002.docx]

**Rebuttal letter Revision 2**

We thank the reviewer for his additional comments. Our answers are in blue below. Please note that with the previous revision, we uploaded the dataset on OSF: <https://osf.io/zxdjs/>.

Reviewer #1: Query 1. Please elaborate your response in the introduction.

We have added the following sentences to the introduction, lines 119-124:

‘While the historic drop in routine childhood immunization coverage in the world is likely due to a myriad of factors, attitudes towards COVID-19 vaccines as well as COVID-19 vaccination status may have played a role (UNICEF 2021). A recent study (not yet peer-reviewed) points to the potential spillover effect from the COVID-19 pandemic, whereby COVID-19 vaccination status and attitudes negatively influence attitudes and vaccination intentions of other vaccines (Trujillo 2022).’

Query 2. Please make a disclaimer that the validation has been carried out and will be published elsewhere. Also, please provide us the non-published supplementary file for the questionnaire validation.

We have added to the Methods, lines 172-176:

‘The Vaccine Trust Indicator (VTI) comprised six items around various aspects of vaccine trust. For instance, about trust in vaccine manufacturers and pharmaceutical companies, trust in the Ministry of Health and understanding how vaccination helps to fight infectious diseases. The scale has been validated and is described in more detail elsewhere (Ellingson 2022).’

As the Ellingson paper is currently under submission elsewhere, we are not able to add the validation results in this paper as a supplement.

Query 3. Please elaborate this (occupation as participants’ characteristic) as the research weakness since we cannot conclude whether the participants are general public or probably they are predominated by health care workers.

Thank you for highlighting this. We have added a sentence in the Strength & Limitations section of the Discussion on this:

‘Furthermore, unmeasured confounders, such as occupation, may have influenced the results.’ (Line 370-371).

We do note that CloudResearch is a market research company that has provided reliable and representative data on the US general public. Their data collection methodology can be found here: <https://go.cloudresearch.com/knowledge/how-does-turkprime-know-panel-demographic-data>

For instance, the paper by Malik et al (see: <https://www.sciencedirect.com/science/article/pii/S258953702030239X> ), predicted that 67% of the US general public would get vaccinated against COVID-19, *before* vaccines were introduced, based on a dataset generated through CloudResearch. That number has held up very well in the real world, demonstrating the generalizability of the data.

Query 6. Please explain why vaccination is considered a healthy lifestyle aspect in the paper.

This statement is part of the Vaccine Trust Indicator, which has been validated elsewhere, as noted under query 2. It is beyond the scope of this paper to describe the reasons why this particular statement was included in the scale. As described in our previous revision, we can assume that people have more positive attitudes towards vaccination when they perceive vaccination to be part of a healthy lifestyle.

Additional Query. Since it is lacking, please enrich the introduction with explanations about the MPXV vaccine, example of the reference: Ophinni et al. 2022. Narra J 2(3): e90 DOI: 10.52225/narra.v2i3.90

Thank you for this recommendation. This citation was already in our reference list, please see reference number 13 and relevant text as below, line 86-109:

‘Fortunately, and contrary to the first year of the COVID-19 pandemic, there are vaccines available against Monkeypox: second and third generation smallpox vaccines.(12-15) The third generation vaccine, the Modified Vaccinia Ankara-Bavarian Nordic vaccine (JYNNEOS), is the primary vaccine being used in the current outbreak in the U.S.(16) At the start of the 2022 global outbreak, their real-world effectiveness was not firmly established, as data were based on observational studies of first-generation smallpox vaccines in the Democratic Republic of the Congo in the 1980s.(14,17) As the 2022 outbreak progressed, real-world vaccine performance data suggest that the JYNNEOS vaccines offers some protection against Monkeypox.(17,18) For instance, in the U.S., the incidence was 14 times higher in unvaccinated males compared to males who had received at least one dose of the JYNNEOS vaccine.(18) In the U.S., the current vaccination strategy includes post-exposure prophylaxis for people who are known contacts to someone with Monkeypox. The vaccine is recommended as pre-exposure prophylaxis for those with higher risk of exposure to Monkeypox, for instance MSM.(19)’
